# Molecular Chaperones: Molecular Assembly Line Brings Metabolism and Immunity in Shape

**DOI:** 10.3390/metabo10100394

**Published:** 2020-10-03

**Authors:** Haoxin Zhao, Lydia N. Raines, Stanley Ching-Cheng Huang

**Affiliations:** 1 Department of Pathology, Case Western Reserve University School of Medicine, Cleveland, OH 44106, USA; hxz619@case.edu (H.Z.); lxr220@case.edu (L.N.R.); 2 Case Comprehensive Cancer Center, Case Western Reserve University School of Medicine, Cleveland, OH 44106, USA

**Keywords:** molecular chaperones, heat shock proteins, environmental stress, immune responses, immunometabolism, epigenetics

## Abstract

Molecular chaperones are a set of conserved proteins that have evolved to assist the folding of many newly synthesized proteins by preventing their misfolding under conditions such as elevated temperatures, hypoxia, acidosis and nutrient deprivation. Molecular chaperones belong to the heat shock protein (HSP) family. They have been identified as important participants in immune functions including antigen presentation, immunostimulation and immunomodulation, and play crucial roles in metabolic rewiring and epigenetic circuits. Growing evidence has accumulated to indicate that metabolic pathways and their metabolites influence the function of immune cells and can alter transcriptional activity through epigenetic modification of (de)methylation and (de)acetylation. However, whether molecular chaperones can regulate metabolic programs to influence immune activity is still largely unclear. In this review, we discuss the available data on the biological function of molecular chaperones to immune responses during inflammation, with a specific focus on the interplay between molecular chaperones and metabolic pathways that drive immune cell fate and function.

## 1. Introduction

Molecular chaperones play a pivotal role in the maintenance of cellular proteostasis by preventing the misfolding and aggregation of nascent polypeptides by ensuring proper protein folding [1,2]. Although constitutively expressed under steady-state, many chaperones are up-regulated by cellular stressors including high temperature. Thus, they were initially identified as heat shock proteins (HSPs). Based on their molecular weight, these proteins have been classified into six major families including HSP100, HSP90, HSP70, HSP60, HSP40 and small heat shock proteins (sHSPs). In addition to preventing pathological aggregation of target proteins/peptides, growing reports have proposed that molecular chaperones can function as intracellular signals to regulate immunity and inflammation [3]. Secreted and extracellular chaperones are able to act as ligands to bind with specific receptors on immune cells, allowing for participation in immune activities such as antigen presentation, immune cell activation and immunomodulation [4,5]. HSPs, therefore, are indicated as active players in both innate and adaptive immunity and have been evaluated as therapeutic targets in a variety of human diseases.

Immune cells are highly heterogeneous in population and dynamic in functional activities. They adopt distinct metabolic states to support both the energetic and biosynthetic demands of a range of processes [6]. In general, as they respond to immunological stimulations, pro-inflammatory immune cells such as M1 macrophages, activated dendritic cells (DCs), neutrophils and effector Th1 cells adopt aerobic glycolysis (also known as the Warburg effect) to support rapid ATP production and the biosynthetic process. In contrast, other immune cells like M2 macrophages, regulatory T cells (Tregs), and memory T cells employ oxidative phosphorylation (OXPHOS) and fatty acid oxidation (FAO) to meet their energy demands [7,8,9]. Recent evidence has shown that certain cellular metabolism-derived metabolites have cytokine-like (e.g., succinate, itaconate) and epigenetic roles (e.g., alpha-ketoglutarate (α-KG) and acetyl coenzyme A (acetyl-CoA)) in immune responses [10,11], highlighting the role of metabolic adaptation in modulating immunity in human diseases such as infection, cancer and inflammatory disorders.

Molecular chaperones have been widely investigated in regard to cancer metabolism and the inflammatory microenvironments, where they act as signals to regulate immunity and inflammation. Several studies have demonstrated that molecular chaperones could affect epigenetic variation to influence numerous transcriptional programs contributing to the phenotypic plasticity of cells [12,13]. The interplay between metabolism and epigenetic adaptions has been suggested to support immune cell homeostasis [11,14,15]. However, the roles of HSPs in metabolic and epigenetic reprogramming of immune cell function and the underlying mechanisms are still unclear. Understanding how these signaling networks come together may ultimately result in new therapeutic approaches to target molecular chaperones and/or down-stream checkpoints in controlling immune cell fate for better disease treatment.

## 2. Overview of Molecular Chaperones

In the early 1960s, Ferruccio Ritossa observed the formation of new puffs in the chromosomes of *Drosophila* larvae upon exposure to increased temperature [16]. In 1970, these proteins were given the name heat shock proteins by Alfred Tissières and colleagues [17]. Later, it became apparent that other stressors such as chemicals, toxins, low pH and oxidative stress could induce these proteins [18]. A subsequent study indicated that there existed a relationship between this heat-induced gene transcript and protein folding during stress [19,20]; however, it would not be until the late 1980s when these proteins were found to be required for the proper folding of cellular proteins [20]. Now, more so than just heat shock induced proteins, it has been found that HSPs are a dynamic range of proteins that share a conserved sequence and structure across all organisms and are induced upon stress stimulation. Based on molecular weight, they are categorized in six major families including HSP100 (100 kDa or higher), HSP90 (83–90 kDa), HSP70 (70 kDa), HSP60 (close to 60 kDa), HSP40 (40 kDa) and small heat shock proteins (sHSPs, 10–30 kDa) [21]. Often, as a consequence of cellular stress, misfolded proteins arise and accumulate. HSPs assist in the folding of unfolded and misfolded proteins by stabilizing folding intermediates to prevent protein misfolding and aggregation [22]. Indeed, while basal expression of HSPs can be seen in normal cells, their expression level is significantly increased upon exposure to intracellular or extracellular stresses, leading to a physiological response known as the heat shock response (HSR) in cells [23]. HSR occurs universally from bacteria to mammals and is regulated by the transcription factor heat shock factor (HSF) [24]. There are four different transcription factors (HSF1-4) in mammalian cells in which HSF1 is considered as the main regulator of HSPs transcription. Structurally, HSF1 includes an N-terminal DNA binding domain and C-terminal DNA transactivation domain in which HSF1 controls HSP gene expression by binding to the heat shock elements (HSEs). In non-stress states, the transcriptional activity of HSF1 is restricted in the cytoplasm by binding to a complex containing HSP70 and HSP90. Upon activation, the chaperone complex dissociates and HSF1 is liberated, allowing it to homotrimerize and translocate into the nucleus. It binds to HSEs located upstream to HSP gene promoters and initiates target HSP gene transcription [25].

In general, stress proteins are believed to act as intracellular molecular chaperones expressed in the cytosol and organelles such as mitochondria, endoplasmic reticulum (ER) and nucleus. Under certain circumstances, they are released to extracellular space and can serve as danger signals, directly stimulating innate immune cells. Indeed, HSP60 and HSP70 have been revealed to bind to toll-like receptor (TLR)2 and TLR4 to activate the downstream signaling pathway for eliciting innate immune responses [26,27]. In addition to triggering innate immunity, the covalent conjugation of antigenic proteins/peptides to HSPs and the generation of HSP fusion proteins such as HSP70, can generate efficient antigen-specific T cell immunity [28,29] and has been employed in vaccine design for infectious diseases and cancers [30]. The uptake of the HSP-peptide complex may involve receptors expressed on the surface of antigen presenting cells (APCs) such as CD91 and can undergo antigen presentation through major histocompatibility complex (MHC) class I and class II pathways [31]. Numerous lines of evidence have indicated the regulatory role of HSPs in immune activity and various immunopathologies including cancer, infection, diabetes, autoimmune and inflammatory diseases [5].

## 3. Roles of Molecular Chaperones in Metabolism and Immune Function

Metabolic pathways are intimately linked to immune function [6,32,33]. Studies have indicated that the differentiation and activation of immune cells requires tight regulation of gene expression governed by epigenetic programs [34,35]. Increasing evidence has suggested that nutrient uptake and metabolic flux can affect epigenetic modifications instructing the function of immune cells [11,36,37]. The activity of most epigenetic enzymes involved in chromatin and DNA modifications is dependent on metabolite availability such as acetyl-CoA, α-KG, *S*-adenosylmethionine (SAM) and nicotinamide adenine dinucleotide (NAD^+^). For instance, the modification of DNA and histone methylation requires SAM metabolite as the methyl donor used by the DNA methyltransferases (DNMTs) and histone methyltransferases (HMTs) in cells, respectively. In contrast, the regulation of demethylation is tailored by ten-eleven translocation (TET) enzymes and histone demethylases (HDMs) using α-KG as a cofactor [38,39]. Numerous reports have suggested that molecular chaperones such as the HSP90 and HSP70 family members can regulate metabolic pathways in response to environmental changes, although most reports focus on cancer cells. In the following sections, we overview the key roles of HSP90, HSP70, HSP60 and HSP40 and small HSP family members in the control of cellular metabolism, inflammation and epigenetic modification. We also discuss the potential connection between HSPs and metabolic reprogramming in immune cells.

### 3.1. HSP90

The members of the HSP90 family are highly conserved proteins that are localized in different cellular compartments. Two HSP90 isoforms, HSP90α (inducible form) and HSP90β (constitutively expressed form) are localized in the cytosol. Other family members including glucose-regulated protein 94 (GRP94) are localized in the ER, while tumor necrosis factor receptor-associated protein 1 (TRAP1) is mainly localized in the mitochondria [40,41]. HSP90 family members are the most abundant molecular chaperones that promote the folding of newly synthesized proteins. Structurally, HSP90 chaperones are constituted by an N-terminal domain (NTD) that binds ATP, a C-terminal domain (CTD) containing subcellular localization sequences and a middle domain (MD) that interacts with client proteins and provides structural flexibility [40,42,43]. In addition to protein folding, HSP90 proteins participate in multiple cellular processes including protein assembly and degradation, cell cycle control, apoptosis and signal transduction and transcriptional regulation [44].

HSP90 chaperones have been shown to induce innate and adaptive immunity by stimulating APCs and facilitating the activation and maturation of lymphocytes [45]. Secreted and extracellular HSP90 can bind to substrate peptide antigens and interact with surface receptors to facilitate antigen uptake by endocytosis in myeloid cells [46]. Intracellular HSP90 is involved in cytosolic translocation of extracellular antigen for cross-presentation by DCs [47]. In line with this, it has been shown that intracellular HSP90 can mediate DC maturation and enhance antigen presentation and T cell activation [48]. Moreover, extracellular HSP90 is thought to facilitate the folding of receptors on T cells and natural killer (NK) cells, thus aiding to promote the full stimulation of these cells [49]. Simultaneously, extracellular or surface HSP90 can serve as a danger/damage-associated molecular pattern (DAMP), which drives innate immune responses including cytokine secretion (e.g., IL-12) and co-stimulatory molecules expression (CD80/CD86), and also T cell activation [50]. HSP90 also has vital roles in regulating the expression of inflammatory genes through the folding of IκB kinase (IKK) and Janus kinase (JAK), which are essential for the activation of nuclear factor-κB (NF-κB) and signal transducer and activators of transcription (STAT) [51,52]. Thus, HSP90 has emerged as a signal to activate immune cells to play proinflammatory roles and is a potential target for inflammatory disease. Indeed, HSP90 inhibition with 17-dimethylaminoethylamino-17-demethoxygeldanamycin (17-DMAG) in vivo reduces pro-inflammatory cytokine TNF-α and prevents lipopolysaccharides (LPS)-induced liver injury, likely via repressive action of HSF1 [53]. Treatment with HSP90 inhibitor can restrict macrophage inflammation and protect from diabetes-associated atherosclerosis via induction of nuclear factor erythroid-derived 2-like 2 (Nrf2) and concomitant inhibition of nuclear factor kappa B (NF-κB)-dependent cytoprotective mechanisms [54]. A recent study indicated that inhibiting the ATPase activity of HSP90 can impair the inflammasome response and can be a candidate for treating inflammasome-mediated diseases [55]. In addition, HSP90 inhibition can prevent caspase-1 (CASP-1) activity and Gasdermin D (GSDMD) and then decreases NLRP3 inflammasome activity and reduces the secretion of cytokines [56]. Conversely, in the tumor microenvironment (TME), tumor-derived HSP90 chaperone complex can stabilize macrophage migration inhibitory factor (MIF) and thereby promote breast tumor progression [57]. Inhibition of HSP90α was shown to promote anti-tumor immunity by reversing multi-modal resistance and the stem-like properties of immune-refractory tumors [58]. Thus, the regulation of immune responses mediated by HSP90 chaperones is distinct and context-dependent.

In past years, numerous reports have suggested that HSP90 chaperones can control metabolic rewiring in cancer cells for tumorigenesis [59,60,61]. HSP90 was found to control the expression of voltage-dependent anion channels (VDAC), a mitochondrial outer membrane protein that interacts with hexokinase II (HKII) contributing to the enhanced capacity of Warburg glucose metabolism in tumor cells. It was then found that inhibition of HSP90 led to a dissociation of HKII from VDAC and the consequent inhibition of glycolytic metabolism and mitochondrial activity [62,63]. Furthermore, transcription factor c-Myc has been reported to play a crucial role in regulating glycolytic metabolism in tumor cells [64] and T lymphocytes [65], and it has been shown that HSP90 can interact with c-Myc, mediating its protein stability to prevent proteolytic degradation. In this case, the inhibition of HSP90 suppressed the expression of c-Myc, leading to a reduction in tumor progression [66,67].

**Figure 1:**
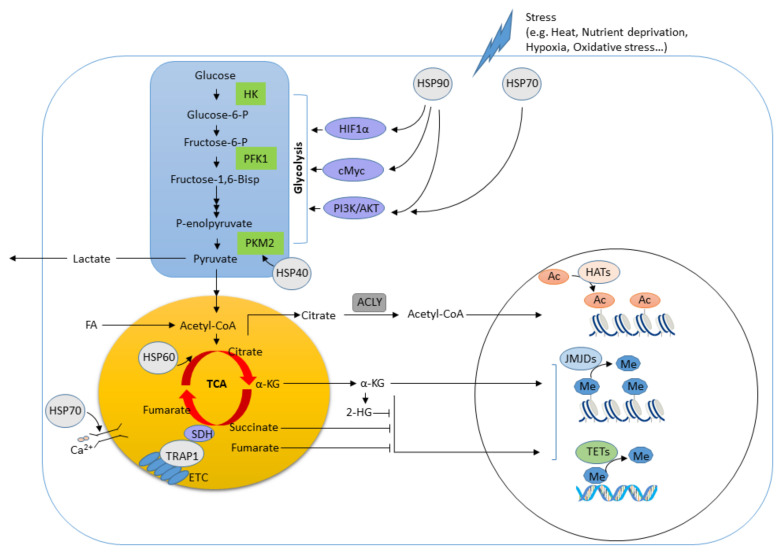
The regulation of molecular chaperones in metabolic pathways and epigenetic modifications. Heat shock proteins (HSPs) can interact with the signaling of HIF1α, cMyc, PI3K/AKT and/or modulate the activity of metabolic enzymes (i.e., HK, PFK1, PKM2 and SDH) to regulate metabolic pathways. The production of metabolites such as acetyl-CoA, α-KG, succinate and fumarate are linked to the function of HATs, JMJDs and TETs involved in epigenetic modification. 2-HG, 2-Hydroxyglutarate; AC, acetylation; ACLY, ATP citrate lyase; α-KG, α-ketoglutarate; AKT, protein kinase B (also known as PKB); ETC, electron transport chain; FA, fatty acid; HATs, histone acetyltransferases; HIF1α, hypoxia-inducible factor 1α; HK, hexokinase; HSP, heat shock protein; JMJDs, Jumonji C domain-containing histone demethylases; Me, Methylation; PFK1, phosphofructokinase 1; PI3K, Phosphoinositide 3-kinase; PKM2, pyruvate kinase 2; SDH, succinate dehydrogenase; TETs, ten-eleven translocation hydroxylases.

Moreover, HSP90 has been demonstrated to interact with and stabilize hypoxia-inducible factor 1-alpha (HIF-1α) [68,69] (Figure 1). HIF-1α is a crucial component of pathways involved in the control of cell metabolism and has a role in regulating immune function of DCs, macrophages, and effector T cells [70,71,72]. AKT (protein kinase B) is an important kinase that supports cell survival, proliferation and metabolism. The interaction between HSP90 and AKT has been found to prevent the proteasomal degradation of AKT and contributes to the functional stabilization of Phosphoinositide 3-kinase (PI3K)/AKT signaling which is vital for glycolysis-dependent survival and activation in T cells [73,74]. Additionally, it has been revealed that HSP90 can directly interact with pyruvate kinase M2 (PKM2), a rate-limiting enzyme of glycolysis, regulating its abundance via Thr-328 phosphorylation to promote tumor cell glycolysis and growth in hepatocellular carcinoma (HCC) [61]. PKM2 is known to translocate into the nucleus of cancer cells to stabilize the transcription factor HIF-1α and to favor the expression of genes associated with glycolysis [75], which has been reported to play critical roles in inflammatory macrophage polarization as well as CD4^+^ T cell activation [76,77]. However, the molecular mechanism of HSP90 signaling linking cellular metabolism and effector function in immune cells remains unclear.

It has been suggested that HSP90 activity may influence phenotypic plasticity through epigenetic modification [12]. Notably, it is known that cellular metabolic alterations and metabolite levels can affect the activity of epigenetic enzymes for modifying distinct transcriptional patterns in cells [11,15,78]. The translocation of PKM2, a client protein of HSP90, promotes the phosphorylation of histone H3 which subsequently removes histone deacetylase 3 (HDAC3), promoting histone H3 acetylation and subsequent gene transcription [79]. Also, PKM2 can directly increase phosphorylation of STAT3 to enhance its transcriptional activity [80]. As mentioned above, nuclear PKM2 binds to HIF-1α to promote the recruitment of HIF-1α and p300 to the promoters of HIF-1α-regulated genes for expression [75]. The activity of HSP90 seems to favor glycolysis in cancer cells, thus supplying increased intermediates such as lactate and acetyl-CoA, both of which can regulate gene expression by epigenetic modification [81,82,83]. Thus, HSP90 may orchestrate the crosstalk between metabolism and epigenetics in controlling the development and function of immune cells [11].

TRAP1, a mitochondrial heat shock protein in the HSP90 family, controls a myriad of physiological roles, including cell proliferation, differentiation and survival [84,85,86]. It has been shown that TRAP1 can mediate the metabolic switch from oxidative phosphorylation (OXPHOS) to glycolysis in cancer cells [84,87]. Briefly, TRAP1 inhibits OXPHOS by interacting with complex II (known as succinate dehydrogenase, SDH) and/or complex IV of the mitochondrial respiratory chain, leading to an accumulation of intracellular succinate (Figure 1). The accumulated succinate inhibits the activity of prolyl-hydroxylase (PHD), which in turn stabilizes HIF-1α to promote tumorigenesis. Mitochondrial respiration is responsible for the production of reactive oxygen species (ROS), and inhibition of complex activity by TRAP1 reduces ROS levels and protects cells from apoptosis, indicating the oncogenic function of TRAP1 [88]; however, others have demonstrated that TRAP1 can act as an oncosuppressor [89]. This suggests that TRAP1 as a modulator of metabolism seems to be context- and tumor-specific [85]. While most reports agree that TRAP1 is involved in metabolic regulation, its role in metabolic reprogramming and effector function in immune cells is still unclear.

### 3.2. HSP70

Chaperone proteins of the HSP70 family are found in all organisms from archaebacteria and plants to humans. The human HSP70 family includes at least eight members. HSP70 protein 5 (HSP70-5, also known as Bip or GRP78) and HSP70-9 (known as mtHSP70 or GRP75) can be found in the ER lumen and the mitochondrial matrix, respectively, and the remaining six HSP70 proteins in the cytosol and nucleus [90,91]. HSP70 has been considered a master molecular chaperone in protein homeostasis and provides a physical platform for the binding of client proteins, other chaperones and co-chaperones [92]. HSP70 was initially believed to be an intracellular chaperone; however, further studies have shown that HSP70 can be secreted under stress [93]. Extracellular HSP70 can act as an inflammatory mediator recognized by pattern recognition receptors (PRRs) to activate the innate immune system, where HSP70 elicits the Toll/Interleukin-1 receptor (TIR) signal pathway by engaging with TLR2 and TLR4 in macrophages [27,94]. Also, exogenous HSP70 released from necrotic cells can bind to TLR7 of macrophages and subsequently activate PI3K and the p38 MAPK signaling cascade, leading to enhanced phagocytosis [95]. In addition, extracellular HSP70 has anti-inflammatory potential which was shown to interact with TLR2 to activate MyD88 and downstream ERK signaling to promote the cytokine production of IL-10 in DCs [96]. Moreover, the treatment of HSP70 reduces the expression of MHCII, CD80 and CD86 in DCs [97] and also impairs the production of TNF-α and IFN-γ in monocytes during inflammation [98]. Similarly, intracellular HSP70 was found to be induced in macrophages upon stimulation for inflammation resolution [99,100,101]. It has been reported that intracellular HSP70 inhibits LPS-induced NF-κB signaling axis through interaction with TRAF6 to block the production of inflammatory mediators (e.g., iNOS and TNF-α) in macrophages [102,103]. Induction of HSP70 mediated by HSF1 can inhibit TLR4-MyD88 signaling cascade and TNF-α production, which is essential for macrophages to cope with alcohol-induced endotoxin tolerance [99], suggesting the anti-inflammatory effect of HSP70.

Recently, it has been found that HSP70 exerts distinct expression patterns in tissue specific macrophages including microglia, monocytes, peritoneal macrophages and adipose tissue macrophages (ATMs) upon metabolic stress [104]. Specifically, HSP70 expression is markedly down-regulated in ATMs but not in other tissue-resident macrophages upon high-fat diet (HFD), which leads to a reduction of the HSP70-mediated anti-inflammatory response and promotes NLRP3 and IL-1β-mediated inflammasome activity [104]. Given the well-described properties of HSP70 in anti-inflammation, an HSP70 activator BGP-15 exhibits a positive effect on metabolic disorders, including diabetes and obesity-induced insulin resistance [105,106,107]. However, the immunomodulation of HSP70 is cell type- and milieu-dependent. It has been illustrated that HSP70 can also stimulate the production of pro-inflammatory cytokines TNF-α, IL-1β, and IL-6 in human monocytes, which is dependent on CD14 and intracellular calcium [108]. Exosomes-containing HSP70 released from mycobacteria-infected macrophages also induce pro-inflammatory function [109]. It has been suggested that HSP70 binds to antigenic proteins and interacts with MHC I on DCs to facilitate cross-presentation, eliciting T cell-mediated immune responses [110,111]. HSP70 is also highly expressed in tumor cells, particularly on the cellular membrane, where it is critical for suppressing senescence to promote tumor growth [112]. In acute myeloid leukemia, membrane HSP70 can be specifically recognized by NK cells via CD94/CD56 to produce granzyme B (GZMB) for cytolytic activity [113,114,115]. In addition, tumor-secreted HSP70 functions as a danger signal to facilitate CD8^+^ T cell infiltration and anti-tumor immunity with macrophages and to suppress tumor growth, highlighting the potential for HSP70-targeted therapy as an efficacious anti-cancer strategy [116].

Further, HSP70 presents immunomodulatory roles in T cells in which HSP70 treatment can elevate the suppressive capacity of Tregs by increasing the production of IL-10 and TGF-β and decreasing the proinflammatory cytokines TNF-α and IFN-γ [117]. In contrast, HSP70 can induce IL-6 production from prostate tissue, which triggers a CD4 and CD8-dependent progressive autoimmune reactivity associated with IL-17 expression and no significant Treg response. This suggests an intimate interconnection between HSP70 and inflammatory responses which can be exploited as immunotherapy for metastatic prostate cancer [118]. Interestingly, environmental cues such as hypoxia, oxidative stress and metabolic stress can promote Th17 differentiation [119], and intracellular HSP70 is also induced in response to such stress conditions, further suggesting a potential link between HSP70 function and immune cell activation/differentiation. In fact, a recent report has shown that under febrile temperature, HSP70 is induced to regulate SMAD4 SUMOylation promoting cell differentiation and effector function of Th17, implicating its critical role in autoimmune diseases [120]. It has been further suggested that microRNAs can interact with HSP70 to increase Th17-associated gene expression [121]. Moreover, a study demonstrated that cryo-thermal therapy to tumor leads to an extracellular secretion of HSP70, in which secreted HSP70 was found to promote the differentiation of myeloid-derived suppressor cells (MDSCs) to mature DCs, contributing to an increased anti-tumor immune response [122].

HSP70 has been shown to control metabolic processes such as mitochondrial respiration, leading to a switch in energy metabolism with a preference towards increased glycolytic metabolism [123]. HSP70 has been reported to regulate AKT signaling to increase glycolytic metabolism in the mouse liver under hypothermic conditions [124], suggesting that HSP70 plays a crucial role in the regulation of metabolic kinases to shape cellular metabolism in response to environmental challenges (Figure 1). The HSP70 family member HSPA12A was found to increase in patients with nonalcoholic steatohepatitis (NASH) and animals fed with a high-fat diet. Additionally, HSPA12A has been shown to act as a novel regulator that interacts with PKM2 in macrophages to control pro-inflammatory M1 activation and cytokine production, leading to hepatocyte steatosis [125]. It has been reported that high temperature exposure can trigger rapid deacetylation of yeast HSP70 (Ssa1) at lysine residues, which modulates respiratory capacity and transcriptional responses [126]. Notably, mitochondrial HSP70 can function at both the ER and mitochondria to orchestrate the regulation of Ca^2+^ signaling between these two organelles and control bioenergetics, cell survival and cell death decisions [127], suggesting its role in regulating the mitochondria–ER crosstalk. Furthermore, reduced expression of HSP70 was found to correlate with DNA hypermethylation mediated by DNA methyltransferase 3A (DNMT3A) in patients with disorders of pseudoexfoliation syndrome and glaucoma [128]. These findings collectively suggest that HSP70 chaperones play pivotal roles in metabolism, inflammation and epigenetics; however, the molecular mechanism by which HSP70 chaperones reprogram metabolic pathways and effector function in immune cells is still unclear and needs further investigation.

### 3.3. HSP60

HSP60 family members are present in the cytosol, mitochondria, and cell surface [129]. HSP60 is initially synthesized in the cytosol where it serves as a response protein for cellular stress. It can subsequently translocate to the mitochondria, where it assists mitochondrial protein folding in an ATP-dependent manner with the co-chaperone HSP10 [130]. Similar to other HSPs, HSP60 proteins have been identified as protective cell stress chaperones or immunological mediators during inflammatory diseases. Evidence indicates that HSP60 provides an interconnection between mitochondrial stress and inflammation, showing that HSP60 expression is elevated during hyperglycemia-induced mitochondrial stress and released from cells to the extracellular environment where it mediates the production of inflammatory factors [131,132]. It has been demonstrated that HSP60 can be secreted from mitochondria into the extracellular environment via exosomes. This allows for the activation of TLR signaling in human neuronal cells and astrocytes during hyperglycemia-induced neuroinflammation [133]. Extracellular HSP60 can also act as a “danger” signal to stimulate the production of co-stimulatory molecules and proinflammatory cytokines (e.g., IL-6, IL-12 and TNF-α), promoting the maturation and activation of DCs and macrophages, respectively [134,135]. Additionally, HSP60 can activate CD14 and p38 MAPK signaling to promote immune activation in human PBMC and monocyte-derived macrophages [4,136]. Besides this, it has been suggested that extracellular HSP60 could possibly bind LPS to facilitate microbe recognition and enhance TLR signaling activity [137]. The activation of APCs by HSP60 subsequently enhances antigen-specific IFN-γ production in T cells to trigger adaptive immunity [138]. HSP60 can also enhance the function of CD4^+^CD25^+^FoxP3^+^ regulatory T cells via innate TLR2 signaling [139]. Moreover, the administration of HSP60 can drive T cell responses that modulate inflammatory diseases in animal models of arthritis, experimental autoimmune encephalomyelitis (EAE), asthma and lupus [140,141]. CD4^+^ T cells from patients with remitting juvenile idiopathic arthritis express higher Th2 marker CD30 responding to HSP60 and can produce regulatory cytokines including IL-10 [142], indicating an important role of HSP60 in the fine balance between promoting and controlling inflammation.

Finally, HSP60 is a quality control protein in the mitochondria and is responsible for maintaining mitochondrial fitness (Figure 1). The dysfunction of HSP60 impairs mitochondrial proteostasis and promotes 5’-AMP-activated protein kinase (AMPK) pathway activity, suppressing the mechanistic/mammalian target of rapamycin complex 1 (mTORC1) signaling and inhibiting cell proliferation in ovarian cancer cells [143]. Studies have shown that HSP60 plays a key role in regulating mitochondrial OXPHOS and subsequently triggers ERK1/2 signaling to promote cell growth in human pancreatic ductal adenocarcinoma (PDAC) [144]. Additionally, HSP60 can mediate the transition between mitochondrial function and glycolysis in clear cell renal cell carcinoma (ccRCC) cancer cells, where suppressed HSP60 promotes Warburg glycolysis and switches mitochondrial function from ATP production to glutamine-directed biosynthesis, thus facilitating tumor progression [145]. Therefore, higher expression of HSP60 is correlated with a better prognosis in patients with clear cell renal cell carcinoma (ccRCC).

### 3.4. HSP40

HSP40 (also known as DnaJ in bacteria) is involved in protein refolding, translocation and degradation, mainly by working as a cofactor of HSP70 [146,147]. HSP40 is involved in bacterial virulence and participates in the host immunomodulation of macrophages. It has been revealed that HSP40 is essential for *Salmonella* to ensure survival within macrophages and invasion of epithelial cells, leading to systemic infection [148]. HSP40 secreted by *Streptococcus pneumoniae* contributes to bacterial survival and elicits innate immune responses by inducing IL-6 production via the PI3K/c-Jun N-terminal kinase (JNK) signaling in macrophages [149]. Based on the immunogenicity of HSP40, it has been considered for vaccine trails against bacterial infection. Previous studies have demonstrated the protective effects of HSP40 against *Streptococcus pneumoniae* infections by promoting the production of IL-10, IFN-γ and IL-17A, as well as increased IgG titers and lymphocyte proliferation in mice in vivo [150,151]. Also, HSP40 has been shown to exert immunoregulatory function in patients with rheumatoid arthritis (RA), where HSP40 represses the proliferation of CD4^+^ and CD8^+^ T cells and stimulates the secretion of IL-10 by PBMCs [152]. In addition, it has been reported that HSP40 can also interact with PKM2 to induce glucose transporter 1 (GLUT1) expression and glucose uptake for regulating glycolysis and proliferation in tumor cells [153,154] (Figure 1).

### 3.5. Small HSPs

Small heat shock proteins (sHSPs) are characterized as small molecular weight proteins between 12 and 43 kDa, containing a core α-crystallin domain (ACD) flanked by variable N-terminal and C-terminal domains and lack an ATPase domain [155]. In general, sHSPs serving as molecular chaperones interact with intermediately folded proteins through surface exposed hydrophobic residues to stabilize the protein and prevent further misfolding and/or aggregation [156]. These interactions hold proteins in a reversible state that helps facilitate refolding or degradation by other chaperones and co-factors. Notably, these interactions require dynamic oligomerization state changes in response to diverse cellular triggers and are ATP-independent [155].

In mammalian cells, ten sHSPs (HSPB1 to HSPB10) have been identified. These are HSPB1 (HSP27), HSPB4 (αA-crystallin), HSPB5 (αB-crystallin), HSPB6 (HSP20) and HSPB8 (HSP22) or Class I sHSPs, and HSPB2, HSPB3, αA-crystallin, HSPB7, HSPB9 and HSPB10 or Class II sHSPs. Class I sHSPs are widely distributed in various tissues and play roles in cell survival during stress, while Class II sHSPs have tissue-specific expression and are involved in tissue development and differentiation [157]. Also, sHSPs exhibit functional activity in the regulation of immune responses. HSPB5 was a potent negative regulator of several inflammatory pathways in both the immune system and the central nervous system (CNS). Hspb5^-/-^ mice exhibit worse EAE symptoms, with higher Th1 and Th17 cytokine secretion from T cells and macrophages and severe CNS inflammation compared with the wildtype controls. However, the administration of recombinant HSPB5 protein ameliorates EAE, suggesting an immunosuppressive and therapeutic role for HSPB5 during autoimmune demyelination [158]. Similarly, HSPB5 was found to regulate intestinal inflammation that inhibits IKKβ-mediated signaling to alleviate colitis symptoms and protect intestinal barrier integrity in animals [159]. The immune balance handled by sHSPs is dependent on their local concentrations. For instance, HSPB5 activates anti-inflammatory activity inducing IL-10 production in macrophages at a lower concentration (around 30 μg/mL). Conversely, high local concentrations of HSP5B were found to develop pro-inflammatory memory T cell responses by producing IFNγ in vitro [160], and importantly, were observed in the brain tissue of multiple sclerosis (MS) patients [161]. It has been shown that HSPB5 and also HSPB4 can bind Cu^2+^ to prevent the generation of ROS and oxidative stress, leading to cytoprotective effects in neurodegenerative diseases [162]. Additionally, HSPB5 can also increase the activity of glucose-6-phosphate dehydrogenase (G6PD) in the pentose phosphate pathway (PPP) [163].

Another class I sHSP HSPB1 can induce the production of IL-10 via activation of p38 signaling in monocytes [164], and exogenous HSPB1 inhibits monocyte differentiation into antigen presenting DCs, suggesting its anti-inflammatory effect [165]. However, HSPB1 also has pro-inflammatory functionality which means it can interact with TLR4 and initiate the downstream signaling cascade to increase the expression of IL-1β and TNF-α in DCs and macrophages [166,167]. Additionally, HSPB1 was shown to exhibit an atheroprotective role by binding to scavenger receptor-A to reduce cholesterol uptake in macrophages and attenuate vascular inflammation [168]. It is known that cholesterol is crucial for programming macrophage immune function especially in the production of inflammatory mediators [169,170] and is mediated by multiple pathways including ER stress signaling [171]. Furthermore, HSPB1 can elevate glutathione (GSH) and ROS levels, which is essential for the protective activity of these proteins against TNFα-induced cell death [172]. For class II sHSP, HSPB2 is involved in inhibiting mitochondrial permeability transition (MPT) via modulating mitochondrial Ca^2+^ uptake, thus decreasing necrosis and apoptosis induced by ischemia/reperfusion (I/R) [173]. Notably, HSPB1 has been reported to be involved in the process of protein refolding in peroxisomes [174]. Peroxisomes are closely connected to the communication between mitochondria and ER through lipid and ROS metabolic pathways, essential metabolites and signals in cellular immune responses [175,176,177]. Polyunsaturated fatty acids (PUFAs) generated by peroxisomes are essential for the synthesis of pro-resolving mediators (e.g., resolvins) to the resolution of inflammation [178,179]. Peroxisomes can regulate cellular cholesterol homeostasis. This metabolic pathway is associated with ER stress signaling mediating anti-tumor immunity in CD8^+^ T cells [180,181]. Also, peroxisomes can control the generation of reactive oxygen species (ROS) and reactive nitrogen species (RNS), mediating cellular redox status and phagosome formation [182,183]. This suggests that sHSPs may coordinate with peroxisomal function to regulate inflammation, metabolism and mitochondria-ER interaction in cells.

### 3.6. Extracellular HSPs

Besides the intracellular functions performed by HSPs, several HSP can be secreted into extracellular space in various pathological conditions. HSP90, HSP70, HSP60, HSP40DnaJ, HSP47 and sHSP as mentioned above have been found outside cells in cancer, neurodegenerative and inflammatory disorders [184,185]. Extracellular (ex)HSPs can serve as danger signals or activators upon stress conditions which are implicated in cell–cell communication and inflammatory responses rather than traditional chaperone activity [186]. It has been reported that exHSP70 can couple with tumor-associated antigens to enhance cross-presentation of APCs and modulate T cell anti-tumor immunity. It has been proposed that this should be used as an HSP-peptide-based vaccine which would lead to tumor repression associated with the generation of specific immunity [187,188]. Moreover, exHSP70 was identified to bind to surface lectin-type oxidized low-density lipoprotein (LDL) receptor-1 (LOX1) on DCs, mediating cross-presentation and leading to CD8^+^ T cell-mediated immunity [189]. Notably, LOX1 signaling is strongly associated with lipid metabolism and histone H4 acetylation for breast cancer molecular phenotypes [190]. LOX1 was found to be highly expressed in macrophages present in human atherosclerotic lesions [191]. exHSP90 (gp96) has been shown to bind to surface receptor CD91 (low-density lipoprotein receptor-related protein 1 (LRP1)) [31] for promoting wound healing [192] and tumor cell invasion [193]. It is known that CD91 is essential for not only phagocytosis, immunosurveillance and Th cell priming [194,195,196], but it is also associated with the regulation of lipid metabolism, glucose homeostasis and inflammation in metabolic syndromes [197]. Collectively, it is likely that exHSPs could act as crucial modulators of metabolism, epigenetics and inflammation in the development and differentiation of immune cells during pathogenesis.

## 4. Concluding Remarks

Molecular chaperones exert a myriad of functions in cells, including protein folding, degradation and transport and protecting proteins from denaturation or aggregation. However, their roles involved in immune stimulation, immune regulation and antigen presentation have attracted much attention. Growing evidence has indicated that cellular metabolism can dictate cell fate and govern the effector function of immune cells via multiple mechanisms including modulating epigenetic networks. It is especially true in fatty acid and amino acid metabolism, where metabolic byproducts fuel epigenetic regulation and transcriptional programs involved in immune cell metabolic reprogramming [39]. The mitochondria and ER play critical roles in protein folding, cellular signaling and metabolic homeostasis that are crucial for the activation and differentiation in immune cells [198,199]. The crosstalk between mitochondria and ER is believed to regulate inflammatory processes during chronic diseases including neurodegeneration, cancer and obesity [200,201,202]. Although molecular chaperones are important for the maintenance of cellular proteostasis, their effects on immune cell metabolism remain unclear. Here, we have pointed out that the function of molecular chaperones is linked to cellular signaling and metabolic pathways in immune cells and could serve as metabolic checkpoints to reprogram immune cell development and function. Ultimately, there is potential for a novel therapeutic target for various pathological insults. It is undoubtedly rapid progress, and more research evidence on the topic of immunometabolism and inflammation will continue to unveil how the delicate balance between host immunity and metabolic circuits is maintained.
